# Supplementary material for: Yoga programme for type-2 diabetes prevention (YOGA-DP) among high risk people in India: a multicentre feasibility randomised controlled trial protocol
Source: BMJ Open. 2020 Sep 6;10(9):e036277. doi: 10.1136/bmjopen-2019-036277 (PMC7477989; doi:10.1136/bmjopen-2019-036277)
Supplement: Supplementary data [file bmjopen-2019-036277supp001.pdf]

Table S1: Structure and content of the Yoga sessions

Yogic practices	Week 1-4<br><br>Each session will last for 45 minutes with the time split as follows:	Week 5+<br><br>Each session should last for 75 minutes with the time split as follows:	Details
Shithilikarana Vyayama	Around 5 minutes	Around 5 minutes	(1) Neck rotation *30 seconds*<br>(2) Shoulder rotation *30 seconds*<br>(3) Elbow flexion and extension *30 seconds*<br>(4) Wrist rotation *30 seconds*<br>(5) Finger movement *30 seconds*<br>(6) Waist rotation *30 seconds*<br>(7) Knee flexion and extension *1 minute*<br>(8) Ankle rotation *1 minute*<br>(9) Toe movement *30 seconds*
Surya Namaskar	--	Around 15 minutes	The below mentioned 12 steps constitute one set of Surya Namaskar. To complete one round of Surya Namaskar, participants need to repeat these 12 steps on the other side of their body (i.e., by extending their left leg behind in step number 4 and bringing their left leg forward in step number 9). Initially, they should practise Surya Namaskar at a slower pace. Only with practice over some time, they may try to do 12 rounds of it at a faster pace for around 15 minutes (i.e., a couple of seconds per step).<br>(1) Pranamasana (prayer pose)<br>(2) Hastauttanasana (raised arms pose)<br>(3) Padahastasana (hands to feet pose)<br>(4) Ashwa Sanchalanasana (equestrian pose)<br>(5) Dandasana (stick pose)<br>(6) Ashtanga Namaskara Asana (salute with eight parts)<br>(7) Bhujangasana (cobra pose)<br>(8) Parvatasana (mountain pose)<br>(9) Ashwa Sanchalanasana (equestrian pose)<br>(10) Padahastasana (hands to feet pose)<br>(11) Hastauttanasana (raised arms pose)<br>(12) Pranamasana (prayer pose)
Asana	Around 15 minutes	Around 30 minutes	Two-sided poses (right and left) are to be practised for about 3 minutes (1.5 minutes on each side) and central-positioned poses are to be practised for about 1.5 minutes. In each session, the Yogic poses are selected from the list below to prevent boredom from the similarity of routine. Advanced Yogic poses are introduced from week 5 onwards, for example, Konasana (angle pose), Trikonasana (triangle pose), Paravakonasana (lateral angle pose), Ardhaustrasana (half camel pose), Ustrasana (camel pose), Dhanurasana (bow pose) and Naukasana (boat pose).<br>(A) Standing poses

			(1) Tadasana (palm tree pose) *1.5 minutes*<br>(2) Ardhachakrasana (half wheel pose) *1.5 minutes*<br>(3) Katichakrasana (waist wheel pose) *3 minutes*<br>(4) Konasana (angle pose) or Trikonasana (triangle pose) or Paravakonasana (lateral angle pose): alternatively *3 minutes*<br>(B) Sitting poses<br>(1) Vajrasana (adamant pose) *1.5 minutes*<br>(2) Mandukasana (frog pose) *1.5 minutes*<br>(3) Ardhaustrasana (half camel pose) or Ustrasana (camel pose): alternatively *1.5 minutes*<br>(4) Vakrasana (twisted pose) or Ardhamatsyendrasana (half spinal twist pose): alternatively *3 minutes*<br>(5) Paschimottanasana (seated forward bend pose) or Janusirsasana (head to knee pose): alternatively *1.5 minutes or 3 minutes, respectively*<br>(C) Lying poses- front/prone<br>(1) Ardhashalabhasana (half locust pose) or Poornashalabhasana (full locust pose): alternatively *3 minutes or 1.5 minutes, respectively*<br>(2) Dhanurasana (bow pose) *1.5 minutes*<br>(3) Makarasana (crocodile pose) *1.5 minutes*<br>(D) Lying poses- back/supine<br>(1) Uttanapadasana (raised legs pose) or Ardhahalasana (half plough pose): alternatively *1.5 minutes*<br>(2) Pavanamuktasana (wind relieving pose) *1.5 minutes*<br>(3) Naukasana (boat pose) *1.5 minutes*<br>(4) Saralmatsyasana (easy fish pose) *1.5 minutes*
Pranayama	Around 13 minutes	Around 13 minutes	(1) Vibhagiya Pranayama (sectional breathing) *4 minutes*<br>(2) Nadishodhana Pranayama (alternate nostril breathing) *3 minutes*<br>(3) Kapalbhati Pranayama (skull shining breathing) or Bhastrika Pranayama (bellow breathing): alternately *3 minutes*<br>(4) Bhramari Pranayama (bee breathing) *3 minutes*
Dhyana and relaxation practices	Around 12 minutes	Around 12 minutes	In each session, the following Dhyana and relaxation practices are to be done in a darkened room.<br>(1) A Kara chanting, U Kara chanting and M Kara chanting *3 minutes*<br>(2) Yoga Nidra (Yogic sleep) *9 minutes*
