# Supplementary material for: Yoga programme for type-2 diabetes prevention (YOGA-DP) among high risk people in India: a multicentre feasibility randomised controlled trial protocol
Source: BMJ Open. 2020 Sep 6;10(9):e036277. doi: 10.1136/bmjopen-2019-036277 (PMC7477989; doi:10.1136/bmjopen-2019-036277)
Supplement: Author's manuscript [file bmjopen-2019-036277.draft_revisions.pdf]

BMJ Open

BMJ Open is committed to open peer review. As part of this commitment we make the peer review history of every article we publish publicly available.

When an article is published we post the peer reviewers' comments and the authors' responses online. We also post the versions of the paper that were used during peer review. These are the versions that the peer review comments apply to.

The versions of the paper that follow are the versions that were submitted during the peer review process. They are not the versions of record or the final published versions. They should not be cited or distributed as the published version of this manuscript.

BMJ Open is an open access journal and the full, final, typeset and author-corrected version of record of the manuscript is available on our site with no access controls, subscription charges or pay-per-view fees (http://bmjopen.bmj.com).

If you have any questions on BMJ Open's open peer review process please email info.bmjopen@bmj.com

BMJ Open

Yoga programme for type-2 diabetes prevention (YOGA-DP) among high risk people in India: a multi-centre feasibility randomised controlled trial protocol

Journal:	*BMJ Open*
Manuscript ID	bmjopen-2019-036277
Article Type:	Protocol
Date Submitted by the Author:	08-Dec-2019
Complete List of Authors:	Chattopadhyay, Kaushik; University of Nottingham<br>Mishra, Pallavi; Centre for Chronic Disease Control<br>Singh, Kavita; Centre for Chronic Disease Control<br>Harris, Tess; St George's University of London<br>Hamer, Mark; University College London<br>Greenfield, Sheila; University of Birmingham<br>Lewis, Sarah; University of Nottingham<br>Manjunath, NK; Swami Vivekananda Yoga Anusandhana Samsthana<br>Nair, Rukamani; Bapu Nature Cure Hospital and Yogashram<br>Mukherjee, Somnath; Bapu Nature Cure Hospital and Yogashram<br>Harper, David; Harper Public Health Consulting Limited<br>Tandon, Nikhil; All India Institute of Medical Sciences,<br>Kinra, Sanjay; London School of Hygiene And Tropical Medicine<br>Prabhakaran, Dorairaj; Centre for Chronic Disease Control
Keywords:	COMPLEMENTARY MEDICINE, DIABETES & ENDOCRINOLOGY, PUBLIC HEALTH, PREVENTIVE MEDICINE

SCHOLARONE™
Manuscripts

I, the Submitting Author has the right to grant and does grant on behalf of all authors of the Work (as defined in the below author licence), an exclusive licence and/or a non-exclusive licence for contributions from authors who are: i) UK Crown employees; ii) where BMJ has agreed a CC-BY licence shall apply, and/or iii) in accordance with the terms applicable for US Federal Government officers or employees acting as part of their official duties; on a worldwide, perpetual, irrevocable, royalty-free basis to BMJ Publishing Group Ltd ("BMJ") its licensees and where the relevant Journal is co-owned by BMJ to the co-owners of the Journal, to publish the Work in this journal and any other BMJ products and to exploit all rights, as set out in our licence.

The Submitting Author accepts and understands that any supply made under these terms is made by BMJ to the Submitting Author unless you are acting as an employee on behalf of your employer or a postgraduate student of an affiliated institution which is paying any applicable article publishing charge ("APC") for Open Access articles. Where the Submitting Author wishes to make the Work available on an Open Access basis (and intends to pay the relevant APC), the terms of reuse of such Open Access shall be governed by a Creative Commons licence – details of these licences and which Creative Commons licence will apply to this Work are set out in our licence referred to above.

Other than as permitted in any relevant BMJ Author's Self Archiving Policies, I confirm this Work has not been accepted for publication elsewhere, is not being considered for publication elsewhere and does not duplicate material already published. I confirm all authors consent to publication of this Work and authorise the granting of this licence.

For peer review only

Title page

Title Yoga programme for type-2 diabetes prevention (YOGA-DP) among high risk people in India: a multi-centre feasibility randomised controlled trial protocol

Authors Kaushik Chattopadhyay[1], Pallavi Mishra[2], Kavita Singh[2], Tess Harris[3], Mark Hamer[4], Sheila Margaret Greenfield[5], Sarah Anne Lewis[1], Nandi Krishnamurthy Manjunath[6], Rukamani Nair[7], Somnath Mukherjee[7], David Ross Harper[8], Nikhil Tandon[9], Sanjay Kinra[10], Dorairaj Prabhakaran[2]; YOGA-DP Study Team

[1]Division of Epidemiology and Public Health, University of Nottingham, UK

[2]Centre for Chronic Disease Control, New Delhi, India

[3]St. George's University of London, UK

[4]University College London, UK

[5]University of Birmingham, UK

[6]Swami Vivekananda Yoga Anusandhana Samsthana, Bengaluru, India

[7]Bapu Nature Cure Hospital and Yogashram, New Delhi, India

[8]Harper Public Health Consulting Limited, London, UK

[9]All India Institute of Medical Sciences, New Delhi, India

[10]London School of Hygiene and Tropical Medicine, UK

Corresponding author Dr Kaushik Chattopadhyay

kaushik.chattopadhyay@nottingham.ac.uk

Abstract word count 298

For peer review only - http://bmjopen.bmj.com/site/about/guidelines.xhtml

Main text word count 2759

For peer review only

For peer review only - http://bmjopen.bmj.com/site/about/guidelines.xhtml

Abstract

Introduction A huge population in India is at high risk of type-2 diabetes mellitus (T2DM). Physical activity and a healthy diet (healthy lifestyle) improve blood glucose levels in people at high risk of T2DM. However, an unhealthy lifestyle is common among Indians. Yoga covers physical activity and a healthy diet and can help to prevent T2DM. The research question to be addressed by the main randomised controlled trial (RCT) is whether a yoga programme for T2DM prevention (YOGA-DP) is effective in preventing T2DM among high risk people in India as compared to enhanced standard care. In this current study, we are determining the feasibility of undertaking the main RCT.

Methods and analysis This is a multi-centre, two-arm, parallel-group, feasibility RCT with blinded outcome assessment and integrated mixed-methods process evaluation. Eligible participants should be aged 18-74 years, at high risk of T2DM (fasting plasma glucose level 5.6 to 6.9 mmol/L) and safe to participate in physical activities. At least 64 participants will be randomised to intervention or control group with final follow-up at six months. Important parameters, needed to design the main RCT, will be estimated, such as standard deviation of the outcome measure (fasting plasma glucose level at 6-month follow-up), recruitment, intervention adherence, follow-up, potential contamination and time needed to conduct the study. Semi-structured qualitative interviews will be conducted with up to 20-30 participants, a sample of those declining to participate, four YOGA-DP instructors and around eight study staff to explore their perceptions and experiences of taking part in the study and of the intervention, reasons behind non-participation, experiences of delivering the intervention and running the study, respectively.

Ethics and dissemination Ethics approval has been obtained from the Research Ethics Committees in India and the UK. The results will be widely disseminated among key stakeholders through various avenues.

Trial registration Clinical Trials Registry- India (CTRI) CTRI/2019/05/018893

For peer review only

Keywords Yoga; physical activity; diet; lifestyle; prevention; prediabetes; blood glucose; feasibility study; randomised controlled trial

Strengths and limitations of this study

- We are determining the feasibility of undertaking the main randomised controlled trial (RCT), and important parameters, needed to design the main RCT, will be estimated.
- This is a multi-centre, two-arm, parallel-group, feasibility RCT with blinded outcome assessment and integrated mixed-methods process evaluation.
- The study is registered with the Clinical Trials Registry- India (CTRI), a part of the World Health Organization (WHO) Registry Network.
- Being a feasibility RCT, it is not adequately powered to detect a difference in outcomes between the two study arms.
- However, appropriate regression methods will be used to get initial estimates of effects with confidence intervals to guide the design of the main RCT.

For peer review only

Introduction

India has the world's second-largest type-2 diabetes mellitus (T2DM) epidemic, a disorder with significant health, social and economic consequences [1]. More than 77 million Indians are in the high risk of T2DM category, with higher blood sugar levels than normal but lower than the established threshold for T2DM itself [2]. They are more likely to develop T2DM and its complications than people with normal blood glucose levels [3]. Physical inactivity and an unhealthy diet are important risk factors of T2DM [3]. Screening of people at high risk of T2DM, followed by an effective lifestyle intervention (i.e., physical activity and a healthy diet) is a cost-effective strategy [3]. It improves blood glucose levels in people at high risk of T2DM and has other health benefits [4,5]. However, physical activity levels are lower among Indians [6]. Similarly, consumption of an unhealthy diet is high among Indians [7,8].

Yoga, an ancient Indian mind-body discipline, covers not only physical activity but also a healthy diet [9]. There are many different styles of yoga, focusing on the same core issue i.e., a healthy lifestyle. No style is necessarily better or more authentic than any other [10]. The acceptability of yoga is usually high among Indians because it fits their health beliefs and culture [11,12]. Generally, yoga uses a gentle approach, is easy to learn and safe, requires a low to moderate level of guidance, is inexpensive to maintain and can be practised indoors and outdoors [11]. It can be practised by older people or those with a wide range of comorbidities - it can help with arthritis and can prevent falls [10,11]. Some of the yogic practices are of low-intensity (<3.5 kcal/min) and some are of moderate-intensity (3.5-7.0 kcal/min) [10,13]. For example, the surya namaskar component of yoga (sun salutation exercises) burns about 3.8-6.7 kcal/min [14,15]. Yoga is also considered as a muscle-strengthening activity [10]. Thus, it can contribute to the aim of routine lifestyle advice to prevent T2DM among high risk individuals.

The beneficial effects of yoga practice on T2DM-related risk profiles appear to occur via two major pathways. First, by reducing the activation and reactivity of the sympathoadrenal system and the hypothalamic-pituitary-adrenal axis, and promoting feelings of well-being, it may

For peer review only - http://bmjopen.bmj.com/site/about/guidelines.xhtml

alleviate the effects of stress and foster multiple positive downstream effects on neuroendocrine status, metabolic function and related systemic inflammatory responses. Second, by directly stimulating the vagus nerve, it may enhance parasympathetic activity and lead to positive changes in cardiovagal function, mood, energy state and in related neuroendocrine, metabolic and inflammatory responses. Furthermore, yoga may lead to weight loss, which itself lowers the risk of T2DM [16].

Systematic reviews of clinical trials suggest beneficial effects of yoga on T2DM-related outcomes in T2DM (as adjuvant therapy) and in metabolic syndrome [17-20]. One such systematic review of 44 randomised controlled trials (RCTs) analysed data from T2DM, metabolic syndrome and healthy participants (n=3168) [17]. Relative to usual care or no intervention, yoga improved blood glucose levels (mean difference=-0.45%; 95% confidence interval=-0.87 to -0.02). No major safety issues were reported. However, most of the included studies were short-term (≤3 months) and were often associated with considerable methodological limitations, such as small sample sizes in treatment groups, resulting in lack of statistical power for outcome assessment, and poor concealment of treatment allocation in outcome assessment, leading to potential analysis bias. Another RCT, conducted recently to prevent T2DM among high risk people in Bengaluru, India, reported similar beneficial effects but had the same methodological issues [21].

Health interventions should be informed by and compatible with the socio-cultural expectations of people and their health beliefs [22]. Yoga is such an intervention in India. The Indian government is committed to and has prioritised the prevention and management of chronic diseases like T2DM through traditional Indian therapies like yoga. The Ministry of AYUSH is dedicated exclusively towards traditional Indian therapies [23]. There is, therefore, a need for a definitive, robustly designed study to assess the utility of yoga in T2DM prevention among high risk people in India. The principal research question to be addressed by the main RCT is whether a yoga programme for T2DM prevention (YOGA-DP) is effective in preventing T2DM among high risk people in India as compared to enhanced standard care. The primary

For peer review only - http://bmjopen.bmj.com/site/about/guidelines.xhtml

outcome of the main RCT will be the difference in mean fasting plasma glucose level between the two treatment arms. We intend to do long-term (≥1 year) follow-ups in the main RCT. The chances of successful completion of a costly T2DM prevention RCT will improve if the feasibility of its key elements is checked before it starts [24,25]. Important parameters, needed to design the main RCT, will be estimated [24]. Thus, in this current study, we are determining the feasibility of undertaking the main RCT.

Methods and analysis

Study design

This is a multi-centre, two-arm, parallel-group, feasibility RCT with blinded outcome assessment and integrated mixed-methods process evaluation.

Study setting

The study is conducted at two yoga centres in India – one each in the northern part of India (Bapu Nature Cure Hospital and Yogashram (BNCHY, New Delhi)) and southern part of India (Swami Vivekananda Yoga Anusandhana Samsthana (S-VYASA, Bengaluru)). Three languages (English, Hindi and Kannada) are used to conduct the study.

Screening and recruitment strategies

A multipronged screening approach is used to identify potential participants:

- Advertisement through posters and pamphlets (placed/distributed at various locations including these yoga centres, communities, religious places, parks and health clinics).
- Screening camps at various locations (including these yoga centres, communities and religious places) and times.
- Door to door visits in various communities and at various times.

After potential participants have been given the participant information sheet, a description of the study and any questions have been answered, people interested in the study are requested to provide written informed consent. Those providing written informed consent are

For peer review only - http://bmjopen.bmj.com/site/about/guidelines.xhtml

assessed against the study eligibility criteria. Their fasting blood glucose level is determined by finger-prick using a glucometer. At these two sites, two glucometer brands are used for this purpose: HemoCue Glucose 201⁺ System and Accu-Chek Active. Those potentially at high risk of T2DM (i.e., fasting blood glucose level 5.6 to 6.9 mmol/L (i.e., 100 to 125 mg/dL)) [26] are invited to these yoga centres for a confirmatory venous blood test, using a standardised method (see Table 1) and after taking further written informed consent. They are re-assessed against the eligibility criteria for the study.

Eligibility criteria

Inclusion criteria

Participants should be:

- Aged 18-74 years.
- At high risk of T2DM.
- Safe to participate in physical activities (assessed using the physical activity readiness questionnaire (PAR-Q)/clinician) [31].
- Willing and able to attend the intervention/control sessions on their own.
- Able to provide written informed consent.

Exclusion criteria

- Pregnant women.
- Those with glycated haemoglobin (HbA1c) ≥6.5% (i.e., ≥48 mmol/mol; with T2DM) [26].
- Those with any serious or uncontrolled medical condition (e.g., cancer).
- Those who regularly practice yoga i.e., ≥150 minutes/week.
- Those currently receiving (or with plans to receive during the study period) any related non-pharmaceutical/pharmaceutical research intervention.

Randomisation

For peer review only

Eligible participants are randomised to intervention or control group according to a computer-generated randomisation schedule (1:1, block randomisation, stratified by sex and site), done centrally by an independent statistician at the Centre for Chronic Disease Control (CCDC), New Delhi, India. This is accessed by calling a telephone line. The exception to this rule is individuals recruited from the same household or if they are close relatives or friends, who are randomised to the same group to avoid contamination. After randomisation, key baseline data are collected. Participants and intervention/control providers cannot be 'blinded' to group allocation but the outcome assessors are 'blind'.

For peer review only

Interventions

Intervention (YOGA-DP)

The YOGA-DP is a structured lifestyle education and exercise programme (see Table 2). The exercise part is based on yoga and includes shithilikarana vyayama (loosening exercises), surya namaskar (sun salutation exercises), asana (yogic poses), pranayama (breathing practices), and dhyana (meditation) and relaxation practices. The intervention has been systematically developed by our study team through reviewing the scientific literature and in consultation with a range of stakeholders (including healthcare, medical and yoga experts and practitioners and the public), which will be published elsewhere. The programme is delivered by YOGA-DP instructors – qualified and experienced yoga teachers with formal training provided on the intervention. Female instructors are available for female participants. Group yoga sessions are run locally (such as at these yoga centres and community centres) at different time points of the day (with evening and weekend sessions), and participants can join as per their convenience. We are reimbursing some of their local travel costs for attending the sessions. A family member or someone close to the participant is invited to join them in the sessions. Once participants complete the programme, they are strongly encouraged to maintain a healthy lifestyle in the long-term, using the intervention booklet and a video.

For peer review only - http://bmjopen.bmj.com/site/about/guidelines.xhtml

Intervention fidelity will be ensured through regular training of YOGA-DP instructors, based on the manual developed for them. Also, sessions will be regularly observed and assessed with a checklist to ensure that they are being delivered as per the manual. To improve performance, structured and instructive feedback will be provided.

Control (enhanced standard care)

Currently, no standard lifestyle intervention is available in India for people at high risk of T2DM. Control group participants will receive a leaflet on routine lifestyle advice to prevent T2DM among high risk individuals. This is delivered by a different team member (i.e., not by the YOGA-DP instructor) to avoid contamination. This provision would ensure that control group participants feel that there are benefits to participation (hence, lower attrition). Contamination could occur in the control group if they start practising yoga during follow-up. However, the specific intervention (YOGA-DP) is not available externally, even if yoga classes are.

Study parameters and data collection

RCT

- Standard deviation (SD) of the outcome measure (fasting plasma glucose level at 6-month follow-up), which will be used to calculate the main RCT sample size.
- Recruitment - number of people approached to participate, written informed consent given, screened for eligibility, found eligible and randomised.
- Intervention adherence - number of sessions attended out of the 27 sessions, number who self-practice at home, and frequency and duration of self-practice at home.
- Follow-up - number of randomised participants followed-up at 6 months.
- Potential contamination - number of control group participants participating in any yoga class during 6-month follow-up (self-reported).
- Time needed to conduct the study (e.g., to recruit participants).
- See Table 1.

For peer review only - http://bmjopen.bmj.com/site/about/guidelines.xhtml

Qualitative evaluation

- Participants: Interviews will be conducted with them to explore their perceptions and experiences of taking part in the study (intervention and control groups participants who complete or do not complete the study) and of the intervention (intervention group participants who complete or do not complete the intervention).
- Those who decline to participate in the study: They are requested to complete a questionnaire (including reasons behind non-participation), and a sample of those who agree to be interviewed to further explore these reasons.
- YOGA-DP instructors and study staff (at the two sites): Interviews will be conducted with them to explore their experiences of delivering the intervention and running the study, respectively.

Pre-developed interview guides will be used by a qualitative researcher to conduct these semi-structured interviews. The interviews will be conducted in interviewees' preferred language and with the help of an interpreter if needed. With consent, these will be noted and digitally-recorded.

Sample size estimation

RCT

At least 64 participants (32/group) will be adequate to precisely estimate the SD of the outcome measure (mentioned before). This is calculated in relation to the desired level of confidence (95%) for the SD, the chosen power (80%) and significance level (5%, two-tailed) of the analysis in the main RCT and the expected loss to follow-up (20% at 6-month) in the current study [32,33].

Qualitative evaluation

For peer review only

- Participants: Interviews will be conducted with up to 20-30 participants. Until data saturation is achieved, purposive sampling will be utilised to ensure the representation of diversity within the RCT population [34].
- Those who decline to participate in the study: A sample of those who agree to be interviewed about their reasons for non-participation, around 10-15 but will continue until saturation is reached [34].
- Four YOGA-DP instructors and around eight study staff (at the two sites).

Data analyses

RCT

Baseline characteristics and important parameters such as follow-up will be summarised and compared between the two study arms using numbers and percentages for categorical data and summary measures of mean or median and spread for continuous data. Being a feasibility RCT, it is not adequately powered to detect a difference in outcomes between the two study arms. However, appropriate regression methods will be used to get initial estimates of effects with confidence intervals to guide the design of the main RCT. All primary analyses will be based on the intention-to-treat principle and will be unadjusted. Subsequently, the adjustment will be done for the respective baseline data and site. No interim analysis is planned.

Qualitative evaluation

All the semi-structured interviews will be transcribed (verbatim), translated (if necessary), anonymised and checked for accuracy. An interpretive analysis will be conducted using thematic analysis, using NVivo software. Transcripts will be read and re-read by two qualitative researchers. These researchers will develop the initial codes and will apply initially to a small number of transcripts, enabling further iteration of the thematic index. We will use illustrative nonattributable quotations [34].

Patient and public involvement

For peer review only

The research topic was identified and discussed with a Public Engagement Coordinator and among a patient and public involvement group. They acknowledged the importance of this research topic and the issues identified during these discussions were taken into consideration while designing the study. They are involved in the discussions and are giving feedback on different aspects of the study.

Ethics and dissemination

Ethics and other related issues

Ethics approval has been obtained from the following Research Ethics Committees: Faculty of Medicine and Health Sciences, University of Nottingham (UK); CCDC (India); BNCHY (India) and S-VYASA (India). We have also received approval from the Health Ministry's Screening Committee (HMSC, India). An independent Trial Steering Committee (TSC) is monitoring and providing overall supervision for the study.

Serious adverse events

Like other physical activities, yoga is known to be safe [10]. Information will be collected on serious adverse events (including death) occurring in participants that may be attributed to the interventions. Based on medical and scientific judgement, an independent clinician will determine the relationship of any event to the interventions.

Participant withdrawal

Participants will be withdrawn from the study either at their request or at the discretion of the site investigator e.g., if diagnosed with diabetes (will receive the standard treatment) or if no longer safe to participate in physical activities (determined by PAR-Q/clinician) [31]. They will be made aware that this will not affect their future care. Also, they will be made aware (via the participant information sheet and consent form) that should they withdraw, the data collected to date will not be erased and may still be used in the final analyses.

Dissemination

For peer review only - http://bmjopen.bmj.com/site/about/guidelines.xhtml

The results will be widely disseminated among key stakeholders through various avenues, such as through dissemination meetings and informal discussions with them, presentations at national and international conferences, publications in peer-reviewed open-access journals, and press offices and websites of host institutions.

Discussion

We are now conducting a multi-centre feasibility RCT in India to determine the feasibility of undertaking the main RCT. The study started in May 2019, and we are aiming to finish the study by the end of April 2020. If the feasibility is promising (such as recruitment, randomisation, intervention adherence and follow-up), then the parameters estimated will be used to design the main RCT. Decisions over whether to modify the protocol will be informed by the process evaluation, including the qualitative data.

If the intervention is found to be effective in the main RCT, it will be a low-cost, acceptable and local solution to prevent T2DM among high risk people in India and to become healthier overall. The future clinical, personal and economic burden of T2DM on patients, their families, the health system and the economy will be prevented. The benefits of preventing T2DM may extend to the prevention of its complications. People will be provided with more evidence-based choices for preventing T2DM. The programme will simultaneously empower them to manage their health. Apart than India and neighbouring South Asian countries, yoga is popular or becoming popular in many other countries [35,36]. Given that T2DM prevention is a global concern and costs are a concern everywhere, a low-cost yoga-based T2DM prevention option will be of interest in other countries, particularly in other South Asian countries and in countries with South Asian ethnic minorities.

Declarations

Ethics approval and consent to participate

For peer review only - http://bmjopen.bmj.com/site/about/guidelines.xhtml

Ethics approval has been obtained from the following Research Ethics Committees: Faculty of Medicine and Health Sciences, University of Nottingham (UK); CCDC (India); BNCHY (India) and S-VYASA (India). Written informed consent is obtained from all the participants.

Consent for publication

Not applicable

Availability of data and materials

Not applicable

Competing interests

The authors declare that they have no competing interests.

Funding

The study is funded by the UK's DFID/MRC/NIHR/Wellcome Trust Joint Global Health Trials (MR/R018278/1). The funding agencies have no role in designing the study or in writing the manuscript.

Authors' contributions

KC conceptualised and designed the study with the help of other authors. KC wrote the first draft of the manuscript and other authors contributed significantly to the revision of the manuscript. All authors read and approved the final manuscript.

Acknowledgements

The authors would like to extend thanks to all who have participated in this study and the TSC members.

References

1. International Diabetes Federation (IDF). IDF diabetes atlas. 8th Ed. Brussels: IDF. 2017.

2. Anjana RM, Pradeepa R, Deepa M, et al. Prevalence of diabetes and prediabetes in urban and rural India: phase I results of the ICMR-INDIAB study. Diabetologia. 2011;54:3022-7.
3. National Institute for Health and Care Excellence (NICE). Preventing type 2 diabetes: risk identification and interventions for individuals at high risk. Manchester: NICE. 2012.
4. Gong QH, Kang JF, Ying YY, et al. Lifestyle interventions for adults with impaired glucose tolerance: a systematic review and meta-analysis of the effects on glycemic control. Intern Med. 2015;54:303-10.
5. World Health Organization (WHO). Global strategy on diet, physical activity and health. Geneva: WHO. 2015.
6. Anjana RM, Pradeepa R, Das AK, et al. Physical activity and inactivity patterns in India: results from the ICMR-INDIAB study (Phase-1) [ICMR-INDIAB-5]. Int J Behav Nutr Phys Act. 2014;11:26.
7. Gulati S, Misra A, Sharma M. Dietary fats and oils in India. Curr Diabetes Rev. 2017;13(5):438-43.
8. Minocha S, Thomas T, Kurpad AV. Are 'fruits and vegetables' intake really what they seem in India? Eur J Clin Nutr. 2018;72:603-8.
9. Feuerstein G. The deeper dimensions of yoga: theory and practice. Boston: Shambhala Publications. 2003.
10. National Health Service (NHS). A guide to yoga: exercise. UK: NHS. 2018. https://www.nhs.uk/live-well/exercise/guide-to-yoga/ accessed on 2 Sep 2019.
11. Anderson JG, Taylor AG. The metabolic syndrome and mind-body therapies: a systematic review. J Nutr Metab. 2011;2011:276419.
12. Anjana RM, Ranjani H, Unnikrishnan R, et al. Exercise patterns and behaviour in Asian Indians: data from the baseline survey of the Diabetes Community Lifestyle Improvement Program. Diabetes Res Clin Pract. 2015;107:77-84.
13. Centers for Disease Control and Prevention (CDC) and American College of Sports Medicine (ACSM). General physical activities defined by level of intensity. Atlanta: CDC. 2015.

For peer review only - http://bmjopen.bmj.com/site/about/guidelines.xhtml

14. Sinha B, Ray US, Pathak A, et al. Energy cost and cardiorespiratory changes during the practice of surya namaskar. Indian J Physiol Pharmacol. 2004;48:184-90.

15. Mody BS. Acute effects of surya namaskar on the cardiovascular and metabolic system. J Bodyw Mov Ther. 2011;15:343-7.

16. Cramer H, Innes KE, Michalsen A, et al. Yoga for adults with type 2 diabetes mellitus (protocol). Cochrane Database Syst Rev. 2015;4:CD011658.

17. Cramer H, Lauche R, Haller H, et al. Effects of yoga on cardiovascular disease risk factors: a systematic review and meta-analysis. Int J Cardiol. 2014;173:170-83.

18. Cramer H, Langhorst J, Dobos G, et al. Yoga for metabolic syndrome: a systematic review and meta-analysis. Eur J Prev Cardiol. 2016;23:1982-93.

19. Kumar V, Jagannathan A, Philip M, et al. Role of yoga for patients with type II diabetes mellitus: a systematic review and meta-analysis. Complement Ther Med. 2016;25:104-12.

20. Cui J, Yan JH, Yan LM, et al. Effects of yoga in adults with type 2 diabetes mellitus: a meta-analysis. J Diabetes Investig. 2017;8:201-9.

21. McDermott KA, Rao MR, Nagarathna R, et al. A yoga intervention for type 2 diabetes risk reduction: a pilot randomized controlled trial. BMC Complement Altern Med. 2014;14:212.

22. Medical Research Council (MRC). Developing and evaluating complex interventions. London: MRC. 2008.

23. Samal J. Situational analysis and future directions of AYUSH: an assessment through 5-year plans of India. J Intercult Ethnopharmacol. 2015;4:348-54.

24. National Institute for Health Research (NIHR). Feasibility and pilot studies. UK: NIHR. 2014.

25. Ranjani H, Weber MB, Anjana RM, et al. Recruitment challenges in a diabetes prevention trial in a low- and middle-income setting. Diabetes Res Clin Pract. 2015;110:51-9.

26. Buysschaert M, Medina JL, Buysschaert B, et al. Definitions (and current controversies) of diabetes and prediabetes. Curr Diabetes Rev. 2016;12:8-13.

27. Craig CL, Marshall AL, Sjöström M, et al. International physical activity questionnaire: 12-country reliability and validity. Med Sci Sports Exerc. 2003;35:1381-95.

For peer review only

28. Herdman M, Gudex C, Lloyd A, et al. Development and preliminary testing of the new five-level version of EQ-5D (EQ-5D-5L). Qual Life Res. 2011;20:1727-36.

29. Henry JD, Crawford JR. The short-form version of the depression anxiety stress scales (DASS-21): construct validity and normative data in a large non-clinical sample. Br J Clin Psychol. 2005;44:227-39.

30. Bandura A. Guide for constructing self-efficacy scales. In: Pajares F, Urdan T (Editors). Self-efficacy beliefs of adolescents. Greenwich: Information Age Publishing. 2006;307-37.

31. Adams R. Revised physical activity readiness questionnaire. Can Fam Physician. 1999;45:992, 995, 1004-5.

32. Hooper R. Justifying sample size for a feasibility study. UK: NIHR. 2014.

33. Dettori JR. Loss to follow-up. Evid Based Spine Care J. 2011;2:7-10.

34. Green J, Thorogood N. Qualitative methods for health research. 3rd Ed. London: SAGE. 2014.

35. Birdee GS, Legedza AT, Saper RB, et al. Characteristics of yoga users: results of a national survey. J Gen Intern Med. 2008;23:1653-8.

36. Ding D, Stamatakis E. Yoga practice in England 1997-2008: prevalence, temporal trends, and correlates of participation. BMC Res Notes. 2014;7:172.

For peer review only

For peer review only - http://bmjopen.bmj.com/site/about/guidelines.xhtml

Table 1: Data collection

	Face-to-face assessments*			
	Assessment details	Screening and recruitment	Baseline	Final at 6-month
**Eligibility assessment**		√		
**Socio-demographics**			√	
**Medical and surgical history**			√	
**Family history of diabetes**			√	
**Current medications**			√	√
**Biochemical parameters^**				
Blood glucose				
Fasting plasma glucose	Glucose oxidase-peroxidase (GOD-POD) method		√	√
Glycated haemoglobin (HbA1c)	High-performance liquid chromatography (HPLC) method		√	√
Lipid profile				
Total cholesterol	Cholesterol oxidase method		√	√
High-density lipoprotein (HDL)	Direct clearance method		√	√
Low-density lipoprotein (LDL)	Direct clearance method		√	√
Very low-density lipoprotein (VLDL)	Calculated value		√	√
Triglyceride	Lipase/Glycerol-3-phosphate oxidase-phenol+aminophenazone (GPO-PAP) no correction method		√	√
**Physiological parameters**				
Blood pressure	Omron HEM-7201		√	√
Heart rate	Omron HEM-7201		√	√
**Anthropometric parameters**				
Waist circumference	Seca 201 (measuring tape)		√	√
Weight	Omron HN-286 (weighing scale)		√	√
Height	Seca 206 (stadiometer)		√	√
Body mass index (BMI)	Calculated value		√	√
**Diet**	Time-recall: past 1-week		√	√

For peer review only

**Physical activity**	International physical activity questionnaire (IPAQ) – short; time-recall: past 1-week[27]		√	√
**Tobacco usage**			√	√
**Alcohol consumption**			√	√
**Health-related quality-of-life**	EuroQol-5D-5L (EQ-5D-5L); time-recall: at the time of questionnaire completion[28]		√	√
**Depression, anxiety and stress**	Depression, anxiety and stress scale (DASS-21); time recall: past 1-week[29]		√	√
**Yoga practice**	Time-recall: past 1-week		√	√
**Self-efficacy** (to assess confidence in participant's ability to practise yoga)	0-100 rating scale; time-recall: at the time of questionnaire completion[30]		√	√

*A standard operating procedure has been developed for this purpose.
^Blood samples are analysed at the International Organization for Standardization (ISO) or Christian Medical College External Quality Assurance Scheme (Vellore, India) accredited laboratories.

For peer review only

For peer review only - http://bmjopen.bmj.com/site/about/guidelines.xhtml

Table 2: Structure of YOGA-DP

Week	Group yoga sessions delivered by YOGA-DP instructors	Self-practice of yoga at home using YOGA-DP booklet and a video	Extra features
1-4 (month 1)	At least two sessions of 45 minutes per week. An attendance register is kept.	--	At the first session, the instructor is giving participants part one of our programme booklet. This gives them information about being at high risk of T2DM and how to prevent T2DM (i.e., by being more physically active, keeping a healthy weight, eating less fat (especially saturated fat) and eating more fibre).
5-12 (month 2-3)	At least two sessions of 75 minutes per week. An attendance register is kept.	--	At the last session, the instructor is giving participants part two of our programme booklet and a video. These give them information on yoga practice to prevent T2DM. Also, a yoga diary and non-slippery yoga mat are provided for self-practice of yoga at home.
13-24 (month 4-6)	At least one session of 75 minutes every four weeks. An attendance register is kept.	At least two sessions of 75 minutes per week. Participants are given the yoga diary to record their yoga practice (types and minutes).	The instructor is phoning participants every week to offer support and help and to troubleshoot any problems.
25+ (month 7+)	--	At least two sessions of 75 minutes per week. Participants are given the yoga diary to record their yoga practice (types and minutes).	--

For peer review only

SPIRIT
STANDARD PROTOCOL ITEMS: RECOMMENDATIONS FOR INTERVENTIONAL TRIALS

SPIRIT 2013 Checklist: Recommended items to address in a clinical trial protocol and related documents*

Section/item	Item No	Description	Addressed on page number
**Administrative information**			
Title	1	Descriptive title identifying the study design, population, interventions, and, if applicable, trial acronym	1
Trial registration	2a	Trial identifier and registry name. If not yet registered, name of intended registry	3
	2b	All items from the World Health Organization Trial Registration Data Set	3
Protocol version	3	Date and version identifier	Not available in web format, please use the contact details to request a copy
Funding	4	Sources and types of financial, material, and other support	15
Roles and responsibilities	5a	Names, affiliations, and roles of protocol contributors	1
	5b	Name and contact information for the trial sponsor	15
	5c	Role of study sponsor and funders, if any, in study design; collection, management, analysis, and interpretation of data; writing of the report; and the decision to submit the report for publication, including whether they will have ultimate authority over any of these activities	15

For peer review only

	5d	Composition, roles, and responsibilities of the coordinating centre, steering committee, endpoint adjudication committee, data management team, and other individuals or groups overseeing the trial, if applicable (see Item 21a for data monitoring committee)	Not available in web format, please use the contact details to request a copy ________
**Introduction**			
Background and rationale	6a	Description of research question and justification for undertaking the trial, including summary of relevant studies (published and unpublished) examining benefits and harms for each intervention	4-6________
	6b	Explanation for choice of comparators	9________
Objectives	7	Specific objectives or hypotheses	4-6________
Trial design	8	Description of trial design including type of trial (eg, parallel group, crossover, factorial, single group), allocation ratio, and framework (eg, superiority, equivalence, noninferiority, exploratory)	6________
**Methods: Participants, interventions, and outcomes**			
Study setting	9	Description of study settings (eg, community clinic, academic hospital) and list of countries where data will be collected. Reference to where list of study sites can be obtained	6________
Eligibility criteria	10	Inclusion and exclusion criteria for participants. If applicable, eligibility criteria for study centres and individuals who will perform the interventions (eg, surgeons, psychotherapists)	7-9________
Interventions	11a	Interventions for each group with sufficient detail to allow replication, including how and when they will be administered	8-9________
	11b	Criteria for discontinuing or modifying allocated interventions for a given trial participant (eg, drug dose change in response to harms, participant request, or improving/worsening disease)	12________
	11c	Strategies to improve adherence to intervention protocols, and any procedures for monitoring adherence (eg, drug tablet return, laboratory tests)	8-9________

For peer review only - http://bmjopen.bmj.com/site/about/guidelines.xhtml

	11d	Relevant concomitant care and interventions that are permitted or prohibited during the trial	7_____________
Outcomes	12	Primary, secondary, and other outcomes, including the specific measurement variable (eg, systolic blood pressure), analysis metric (eg, change from baseline, final value, time to event), method of aggregation (eg, median, proportion), and time point for each outcome. Explanation of the clinical relevance of chosen efficacy and harm outcomes is strongly recommended	9-10,19__________
Participant timeline	13	Time schedule of enrolment, interventions (including any run-ins and washouts), assessments, and visits for participants. A schematic diagram is highly recommended (see Figure)	19-20_____________
Sample size	14	Estimated number of participants needed to achieve study objectives and how it was determined, including clinical and statistical assumptions supporting any sample size calculations	10-11_____________
Recruitment	15	Strategies for achieving adequate participant enrolment to reach target sample size	6_____________

Methods: Assignment of interventions (for controlled trials)

Allocation:

Sequence generation	16a	Method of generating the allocation sequence (eg, computer-generated random numbers), and list of any factors for stratification. To reduce predictability of a random sequence, details of any planned restriction (eg, blocking) should be provided in a separate document that is unavailable to those who enrol participants or assign interventions	7-8_____________
Allocation concealment mechanism	16b	Mechanism of implementing the allocation sequence (eg, central telephone; sequentially numbered, opaque, sealed envelopes), describing any steps to conceal the sequence until interventions are assigned	7-8_____________
Implementation	16c	Who will generate the allocation sequence, who will enrol participants, and who will assign participants to interventions	7-8_____________
Blinding (masking)	17a	Who will be blinded after assignment to interventions (eg, trial participants, care providers, outcome assessors, data analysts), and how	7-8_____________
	17b	If blinded, circumstances under which unblinding is permissible, and procedure for revealing a participant's allocated intervention during the trial	N/A____________

Methods: Data collection, management, and analysis

Data collection methods	18a	Plans for assessment and collection of outcome, baseline, and other trial data, including any related processes to promote data quality (eg, duplicate measurements, training of assessors) and a description of study instruments (eg, questionnaires, laboratory tests) along with their reliability and validity, if known. Reference to where data collection forms can be found, if not in the protocol	19-20
	18b	Plans to promote participant retention and complete follow-up, including list of any outcome data to be collected for participants who discontinue or deviate from intervention protocols	12
Data management	19	Plans for data entry, coding, security, and storage, including any related processes to promote data quality (eg, double data entry; range checks for data values). Reference to where details of data management procedures can be found, if not in the protocol	Not available in web format, please use the contact details to request a copy
Statistical methods	20a	Statistical methods for analysing primary and secondary outcomes. Reference to where other details of the statistical analysis plan can be found, if not in the protocol	11
	20b	Methods for any additional analyses (eg, subgroup and adjusted analyses)	11
	20c	Definition of analysis population relating to protocol non-adherence (eg, as randomised analysis), and any statistical methods to handle missing data (eg, multiple imputation)	11

Methods: Monitoring

Data monitoring	21a	Composition of data monitoring committee (DMC); summary of its role and reporting structure; statement of whether it is independent from the sponsor and competing interests; and reference to where further details about its charter can be found, if not in the protocol. Alternatively, an explanation of why a DMC is not needed	Not available in web format, please use the contact details to request a copy
	21b	Description of any interim analyses and stopping guidelines, including who will have access to these interim results and make the final decision to terminate the trial	N/A
Harms	22	Plans for collecting, assessing, reporting, and managing solicited and spontaneously reported adverse events and other unintended effects of trial interventions or trial conduct	12

Auditing	23	Frequency and procedures for auditing trial conduct, if any, and whether the process will be independent from investigators and the sponsor	Not available in web format, please use the contact details to request a copy
**Ethics and dissemination**			
Research ethics approval	24	Plans for seeking research ethics committee/institutional review board (REC/IRB) approval	11-12
Protocol amendments	25	Plans for communicating important protocol modifications (eg, changes to eligibility criteria, outcomes, analyses) to relevant parties (eg, investigators, REC/IRBs, trial participants, trial registries, journals, regulators)	Not available in web format, please use the contact details to request a copy
Consent or assent	26a	Who will obtain informed consent or assent from potential trial participants or authorised surrogates, and how (see Item 32)	6-7
	26b	Additional consent provisions for collection and use of participant data and biological specimens in ancillary studies, if applicable	Not available in web format, please use the contact details to request a copy
Confidentiality	27	How personal information about potential and enrolled participants will be collected, shared, and maintained in order to protect confidentiality before, during, and after the trial	Not available in web format, please use the contact details to request a copy
Declaration of interests	28	Financial and other competing interests for principal investigators for the overall trial and each study site	14
Access to data	29	Statement of who will have access to the final trial dataset, and disclosure of contractual agreements that limit such access for investigators	Not available in web format, please use the contact details to request a copy

For peer review only

For peer review only - http://bmjopen.bmj.com/site/about/guidelines.xhtml

Ancillary and post-trial care	30	Provisions, if any, for ancillary and post-trial care, and for compensation to those who suffer harm from trial participation	Not available in web format, please use the contact details to request a copy
Dissemination policy	31a	Plans for investigators and sponsor to communicate trial results to participants, healthcare professionals, the public, and other relevant groups (eg, via publication, reporting in results databases, or other data sharing arrangements), including any publication restrictions	14
	31b	Authorship eligibility guidelines and any intended use of professional writers	N/A
	31c	Plans, if any, for granting public access to the full protocol, participant-level dataset, and statistical code	Not available in web format, please use the contact details to request a copy
**Appendices**			
Informed consent materials	32	Model consent form and other related documentation given to participants and authorised surrogates	Not available in web format, please use the contact details to request a copy
Biological specimens	33	Plans for collection, laboratory evaluation, and storage of biological specimens for genetic or molecular analysis in the current trial and for future use in ancillary studies, if applicable	Not available in web format, please use the contact details to request a copy

*It is strongly recommended that this checklist be read in conjunction with the SPIRIT 2013 Explanation & Elaboration for important clarification on the items. Amendments to the protocol should be tracked and dated. The SPIRIT checklist is copyrighted by the SPIRIT Group under the Creative Commons "Attribution-NonCommercial-NoDerivs 3.0 Unported" license.

For peer review only

Yoga programme for type-2 diabetes prevention (YOGA-DP) among high risk people in India: a multi-centre feasibility randomised controlled trial protocol

Journal:	*BMJ Open*
Manuscript ID	bmjopen-2019-036277.R1
Article Type:	Protocol
Date Submitted by the Author:	01-May-2020

Complete List of Authors:	Chattopadhyay, Kaushik; University of Nottingham<br>Mishra, Pallavi; Centre for Chronic Disease Control<br>Singh, Kavita; Centre for Chronic Disease Control<br>Harris, Tess; St George's University of London<br>Hamer, Mark; University College London<br>Greenfield, Sheila; University of Birmingham<br>Lewis, Sarah; University of Nottingham<br>Manjunath, NK; Swami Vivekananda Yoga Anusandhana Samsthana<br>Nair, Rukamani; Bapu Nature Cure Hospital and Yogashram<br>Mukherjee, Somnath; Bapu Nature Cure Hospital and Yogashram<br>Harper, David; Harper Public Health Consulting Limited<br>Tandon, Nikhil; All India Institute of Medical Sciences,<br>Kinra, Sanjay; London School of Hygiene And Tropical Medicine<br>Prabhakaran, Dorairaj; Centre for Chronic Disease Control

**Primary Subject Heading**:	Diabetes and endocrinology
Secondary Subject Heading:	Complementary medicine
Keywords:	COMPLEMENTARY MEDICINE, DIABETES & ENDOCRINOLOGY, PUBLIC HEALTH, PREVENTIVE MEDICINE

SCHOLARONE™
Manuscripts

I, the Submitting Author has the right to grant and does grant on behalf of all authors of the Work (as defined in the below author licence), an exclusive licence and/or a non-exclusive licence for contributions from authors who are: i) UK Crown employees; ii) where BMJ has agreed a CC-BY licence shall apply, and/or iii) in accordance with the terms applicable for US Federal Government officers or employees acting as part of their official duties; on a worldwide, perpetual, irrevocable, royalty-free basis to BMJ Publishing Group Ltd ("BMJ") its licensees and where the relevant Journal is co-owned by BMJ to the co-owners of the Journal, to publish the Work in this journal and any other BMJ products and to exploit all rights, as set out in our licence.

The Submitting Author accepts and understands that any supply made under these terms is made by BMJ to the Submitting Author unless you are acting as an employee on behalf of your employer or a postgraduate student of an affiliated institution which is paying any applicable article publishing charge ("APC") for Open Access articles. Where the Submitting Author wishes to make the Work available on an Open Access basis (and intends to pay the relevant APC), the terms of reuse of such Open Access shall be governed by a Creative Commons licence – details of these licences and which Creative Commons licence will apply to this Work are set out in our licence referred to above.

Other than as permitted in any relevant BMJ Author's Self Archiving Policies, I confirm this Work has not been accepted for publication elsewhere, is not being considered for publication elsewhere and does not duplicate material already published. I confirm all authors consent to publication of this Work and authorise the granting of this licence.

For peer review only

Title page

Title Yoga programme for type-2 diabetes prevention (YOGA-DP) among high risk people in India: a multi-centre feasibility randomised controlled trial protocol

Authors Kaushik Chattopadhyay[1], Pallavi Mishra[2], Kavita Singh[2], Tess Harris[3], Mark Hamer[4], Sheila Margaret Greenfield[5], Sarah Anne Lewis[1], Nandi Krishnamurthy Manjunath[6], Rukamani Nair[7], Somnath Mukherjee[7], David Ross Harper[8], Nikhil Tandon[9], Sanjay Kinra[10], Dorairaj Prabhakaran[2]; YOGA-DP Study Team

[1]Division of Epidemiology and Public Health, University of Nottingham, UK

[2]Centre for Chronic Disease Control, New Delhi, India

[3]St. George's University of London, UK

[4]University College London, UK

[5]Institute of Applied Health Research, University of Birmingham, UK

[6]Swami Vivekananda Yoga Anusandhana Samsthana, Bengaluru, India

[7]Bapu Nature Cure Hospital and Yogashram, New Delhi, India

[8]Harper Public Health Consulting Limited, London, UK

[9]All India Institute of Medical Sciences, New Delhi, India

[10]London School of Hygiene and Tropical Medicine, UK

Corresponding author Dr Kaushik Chattopadhyay

kaushik.chattopadhyay@nottingham.ac.uk

For peer review only

Abstract

Introduction A huge population in India is at high risk of type-2 diabetes (T2DM). Physical activity and a healthy diet (healthy lifestyle) improve blood glucose levels in people at high risk of T2DM. However, an unhealthy lifestyle is common among Indians. Yoga covers physical activity and a healthy diet and can help to prevent T2DM. The research question to be addressed by the main randomised controlled trial (RCT) is whether a Yoga programme for T2DM prevention (YOGA-DP) is effective in preventing T2DM among high risk people in India as compared to enhanced standard care. In this current study, we are determining the feasibility of undertaking the main RCT.

Intervention YOGA-DP is a structured lifestyle education and exercise programme. The exercise part is based on Yoga and includes Shithilikarana Vyayama (loosening exercises), Surya Namaskar (sun salutation exercises), Asana (Yogic poses), Pranayama (breathing practices) and Dhyana (meditation) and relaxation practices.

Methods and analysis This is a multi-centre, two-arm, parallel-group, feasibility RCT with blinded outcome assessment and integrated mixed-methods process evaluation. Eligible participants should be aged 18-74 years, at high risk of T2DM (fasting plasma glucose level 5.6 to 6.9 mmol/L) and safe to participate in physical activities. At least 64 participants will be randomised to intervention or control group with final follow-up at six months. Important parameters, needed to design the main RCT, will be estimated, such as standard deviation of the outcome measure (fasting plasma glucose level at 6-month follow-up), recruitment, intervention adherence, follow-up, potential contamination and time needed to conduct the study. Semi-structured qualitative interviews will be conducted with up to 20-30 participants, a sample of those declining to participate, four YOGA-DP instructors and around eight study staff to explore their perceptions and experiences of taking part in the study and of the intervention, reasons behind non-participation, experiences of delivering the intervention and running the study, respectively.

For peer review only - http://bmjopen.bmj.com/site/about/guidelines.xhtml

Ethics and dissemination Ethics approval has been obtained from the following Research Ethics Committees: Faculty of Medicine and Health Sciences, University of Nottingham (UK); CCDC (India); BNCHY (India) and S-VYASA (India). The results will be widely disseminated among key stakeholders through various avenues.

Trial registration Clinical Trials Registry- India (CTRI) CTRI/2019/05/018893

Keywords Yoga; physical activity; diet; lifestyle; prevention; prediabetes; blood glucose; feasibility study; randomised controlled trial

Strengths and limitations of this study

- We are determining the feasibility of undertaking the main randomised controlled trial (RCT), and important parameters, needed to design the main RCT, will be estimated.
- This is a multi-centre, two-arm, parallel-group, feasibility RCT with blinded outcome assessment and integrated mixed-methods process evaluation.
- The study is registered with the Clinical Trials Registry- India (CTRI), a part of the World Health Organization (WHO) Registry Network.
- Being a feasibility RCT, it is not adequately powered to detect a difference in outcomes between the two study arms.
- However, appropriate regression methods will be used to get initial estimates of effects with confidence intervals to guide the design of the main RCT.

For peer review only

Introduction

India has the world's second-largest type-2 diabetes (T2DM) epidemic, a disorder with significant health, social and economic consequences [1]. More than 77 million Indians are in the high risk of T2DM category, with higher blood sugar levels than normal, but lower than the established threshold for T2DM itself [2]. They are more likely to develop T2DM and its complications than people with normal blood glucose levels [3]. Physical inactivity and an unhealthy diet are important risk factors of T2DM [3]. Screening of people at high risk of T2DM, followed by an effective lifestyle intervention (i.e., physical activity and a healthy diet) is a cost-effective strategy [3]. It improves blood glucose levels in people at high risk of T2DM and has other health benefits [4,5]. However, physical activity levels are lower among Indians [6]. Similarly, consumption of an unhealthy diet is high among Indians [7,8].

Yoga, an ancient Indian mind-body discipline, covers not only physical activity, but also a healthy diet [9]. There are many different styles of Yoga, focusing on the same core issue i.e., a healthy lifestyle. No style is necessarily better or more authentic than any other [10]. The acceptability of Yoga is usually high among Indians because it fits their health beliefs and culture [11,12]. Generally, Yoga uses a gentle approach, is easy to learn and safe, requires a low to moderate level of guidance, is inexpensive to maintain and can be practised indoors and outdoors [11]. It can be practised by older people or those with a wide range of comorbidities - it can help with arthritis and can prevent falls [10,11]. Some of the Yogic practices are of low-intensity (<3.5 kcal/min) and some are of moderate-intensity (3.5-7.0 kcal/min) [10,13]. For example, the Surya Namaskar component of Yoga (sun salutation exercises) burns about 3.8-6.7 kcal/min [14,15]. Yoga is also considered as a muscle-strengthening activity [10]. Thus, it can contribute to the aim of routine lifestyle advice to prevent T2DM among high risk individuals.

The beneficial effects of Yoga practice on T2DM-related risk profiles appear to occur via two major pathways. First, by reducing the activation and reactivity of the sympathoadrenal system and the hypothalamic-pituitary-adrenal axis, and promoting feelings of well-being, it may

For peer review only

alleviate the effects of stress and foster multiple positive downstream effects on neuroendocrine status, metabolic function and related systemic inflammatory responses. Second, by directly stimulating the vagus nerve, it may enhance parasympathetic activity and lead to positive changes in cardiovagal function, mood, energy state and in related neuroendocrine, metabolic and inflammatory responses. Furthermore, Yoga may lead to weight loss, which itself lowers the risk of T2DM [16].

Systematic reviews of clinical trials suggest beneficial effects of Yoga on T2DM-related outcomes in T2DM (as adjuvant therapy) and in metabolic syndrome [17-20]. One such systematic review of 44 randomised controlled trials (RCTs) analysed data from T2DM, metabolic syndrome and healthy participants (n=3168) [17]. Relative to usual care or no intervention, Yoga improved blood glucose levels (mean difference=-0.45%; 95% confidence interval=-0.87 to -0.02). No major safety issues were reported. However, most of the included studies were short-term (≤3 months) and were often associated with considerable methodological limitations, such as small sample sizes in treatment groups, resulting in lack of statistical power for outcome assessment, and poor concealment of treatment allocation in outcome assessment, leading to potential analysis bias.

In addition, some of the relevant previous studies have not described the intervention in detail, making it difficult to replicate successful interventions [17-20]. Most studies have not reported the intervention development process. It is hard to know whether these interventions were carefully thought out (e.g., their safety and acceptability) and comprehensive in their development. Thus, it is difficult to select (and replicate) one successful intervention over another. A further selection barrier is their heterogeneous contents, which needed to be summarised for utilisation in T2DM prevention. Therefore, we addressed these issues by systematically developing a Yoga programme for T2DM prevention (YOGA-DP) among high risk people in India, which will be published elsewhere. Briefly, this iterative process included five steps: (i) a systematic review of the literature to generate a list of Yogic practices that improves blood glucose levels among adults at high risk of or with T2DM, (ii) validation of

For peer review only - http://bmjopen.bmj.com/site/about/guidelines.xhtml

identified Yogic practices by Yoga experts, (iii) development of the intervention, (iv) consultation with a range of relevant experts about the intervention and (v) pretest the intervention among Yoga practitioners and lay people in India.

Health interventions should be informed by and compatible with the socio-cultural expectations of people and their health beliefs [21]. Yoga is such an intervention in India. The Indian government is committed to and has prioritised the prevention and management of chronic diseases like T2DM through traditional Indian therapies like Yoga. The Ministry of AYUSH is dedicated exclusively towards traditional Indian therapies [22]. There is, therefore, a need for a definitive, robustly designed study to assess the utility of Yoga in T2DM prevention among high risk people in India. The principal research question to be addressed by the main RCT is whether YOGA-DP is effective in preventing T2DM among high risk people in India as compared to enhanced standard care. The primary outcome of the main RCT will be the difference in mean fasting plasma glucose level between the two treatment arms. We intend to do long-term (≥1 year) follow-ups in the main RCT. The chances of successful completion of a costly T2DM prevention RCT will improve if the feasibility of its key elements is checked before it starts [23,24]. Important parameters, needed to design the main RCT, will be estimated [23]. Thus, in this current study, we are determining the feasibility of undertaking the main RCT.

Methods and analysis

Study design

This is a multi-centre, two-arm, parallel-group, feasibility RCT (see Figure 1) with blinded outcome assessment and integrated mixed-methods process evaluation.

Study setting

The study is conducted at two Yoga centres in India – one each in the northern part of India (Bapu Nature Cure Hospital and Yogashram (BNCHY, New Delhi)) and southern part of India (Swami Vivekananda Yoga Anusandhana Samsthana (S-VYASA, Bengaluru)). People from

For peer review only

a range of socio-economic backgrounds access the services provided by these two research-active Yoga centres. Three languages (English, Hindi and Kannada) are used to conduct the study.

Sample size estimation

RCT

At least 64 participants (32/group) will be adequate to precisely estimate the standard deviation (SD) of the outcome measure (fasting plasma glucose level at 6-month follow-up). This is calculated in relation to the desired level of confidence (95%) for the SD, the chosen power (80%) and significance level (5%, two-tailed) of the analysis in the main RCT and the expected loss to follow-up (20% at 6-month) in the current study [25,26].

Qualitative evaluation

- Participants: Interviews will be conducted with up to 20-30 participants. Until data saturation is achieved, purposive sampling will be utilised to ensure the representation of diversity within the RCT population [27].
- Those who decline to participate in the study: A sample of those who agree to be interviewed about their reasons for non-participation, around 10-15, but will continue until saturation is reached [27].
- Four YOGA-DP instructors and around eight study staff (at the two sites).

Screening and recruitment strategies

A multipronged approach is used to identify potential participants at both sites:

- Advertisement through posters and pamphlets (placed/distributed at various locations including these Yoga centres, communities, religious places, parks and health clinics).
- Screening camps at various locations (including these Yoga centres, communities and religious places) and times.
- Door to door visits in various communities and at various times.

For peer review only - http://bmjopen.bmj.com/site/about/guidelines.xhtml

After potential participants have been given the participant information sheet, a description of the study and any questions have been answered, people interested in the study are requested to provide written informed consent. Those providing written informed consent are assessed against the study eligibility criteria. Their fasting blood glucose level is determined by finger-prick using a glucometer. At these two sites, two glucometer brands are used for this purpose: HemoCue Glucose 201^{+} System and Accu-Chek Active. Those potentially at high risk of T2DM (i.e., fasting blood glucose level 5.6 to 6.9 mmol/L (i.e., 100 to 125 mg/dL)) [28] are invited to these Yoga centres for a confirmatory venous blood test, using a standardised method (see Table 1) and after taking further written informed consent. They are re-assessed against the eligibility criteria for the study.

Eligibility criteria

Inclusion criteria

Participants should be:

- Aged 18-74 years.
- At high risk of T2DM.
- Safe to participate in physical activities (assessed using the physical activity readiness questionnaire (PAR-Q)/clinician) [29].
- Willing and able to attend the intervention/control sessions on their own.
- Able to provide written informed consent.

Exclusion criteria

- Pregnant women.
- Those with glycated haemoglobin (HbA1c) ≥6.5% (i.e., ≥48 mmol/mol; with T2DM) [28].
- Those with any serious or uncontrolled medical condition (e.g., cancer).
- Those who regularly practice Yoga i.e., ≥150 minutes/week.

For peer review only - http://bmjopen.bmj.com/site/about/guidelines.xhtml

- Those currently receiving (or with plans to receive during the study period) any related non-pharmaceutical/pharmaceutical research intervention.

Randomisation

Eligible participants are randomised to intervention or control group according to a computer-generated randomisation schedule (1:1, block randomisation, stratified by sex and site), done centrally by an independent statistician at the Centre for Chronic Disease Control (CCDC), New Delhi, India. This is accessed by calling a telephone line. The exception to this rule is individuals recruited from the same household or if they are close relatives or friends, who are randomised to the same group to avoid contamination. After randomisation, key baseline data are collected. Participants and intervention/control providers cannot be ‘blinded’ to group allocation, but the outcome assessors are ‘blind’.

Interventions

Intervention (YOGA-DP)

YOGA-DP is a structured lifestyle education and exercise programme, provided over a period of 24 weeks (see Table 2). The exercise part is based on Yoga and includes Shithilikarana Vyayama (loosening exercises), Surya Namaskar (sun salutation exercises), Asana (Yogic poses), Pranayama (breathing practices), and Dhyana (meditation) and relaxation practices. Table S1 shows the structure and content of the Yoga sessions.The programme is delivered by YOGA-DP instructors – qualified and experienced Yoga teachers with formal training provided on the intervention. Female instructors are available for female participants. Group Yoga sessions are run locally (such as at these Yoga centres and community centres) at different time points of the day (with evening and weekend sessions), and participants can join as per their convenience. We are reimbursing some of their local travel costs for attending the sessions. A family member or someone close to the participant is invited to join them in the sessions. Once participants complete the programme, they are strongly encouraged to maintain a healthy lifestyle in the long-term, using the intervention booklet and a video.

For peer review only - http://bmjopen.bmj.com/site/about/guidelines.xhtml

Intervention fidelity will be ensured through regular training of YOGA-DP instructors, based on the manual developed for them. Also, sessions will be regularly observed and assessed with a checklist to ensure that they are being delivered as per the manual. To improve performance, structured and instructive feedback will be provided.

Control (enhanced standard care)

Currently, no standard lifestyle intervention is available in India for people at high risk of T2DM. Control group participants will receive a leaflet on routine lifestyle advice to prevent T2DM among high risk individuals. This is delivered by a different team member (i.e., not by the YOGA-DP instructor) to avoid contamination. This provision would ensure that control group participants feel that there are benefits to participation (hence, lower attrition). Contamination could occur in the control group if they start practising Yoga during follow-up. However, the specific intervention (YOGA-DP) is not available externally, even if Yoga classes are.

Study parameters and data collection

RCT

- SD of the outcome measure (mentioned before), which will be used to calculate the main RCT sample size.
- Recruitment - number of people approached to participate, written informed consent given, screened for eligibility, found eligible and randomised.
- Intervention adherence - number of sessions attended out of the 27 sessions, number who self-practice at home, and frequency and duration of self-practice at home.
- Follow-up - number of randomised participants followed-up at 6 months.
- Potential contamination - number of control group participants participating in any Yoga class during 6-month follow-up (self-reported).
- Time needed to conduct the study (e.g., to recruit participants).
- See Table 1.

For peer review only - http://bmjopen.bmj.com/site/about/guidelines.xhtml

Qualitative evaluation

- Participants: Interviews will be conducted with them to explore their perceptions and experiences of taking part in the study (intervention and control group participants who complete or do not complete the study) and of the intervention (intervention group participants who complete or do not complete the intervention).
- Those who decline to participate in the study: They are requested to complete a questionnaire (including reasons behind non-participation), and a sample of those who agree to be interviewed to further explore these reasons.
- YOGA-DP instructors and study staff (at the two sites): Interviews will be conducted with them to explore their experiences of delivering the intervention and running the study, respectively.

Pre-developed interview guides will be used by a qualitative researcher to conduct these semi-structured interviews. The interviews will be conducted in interviewees' preferred language and with the help of an interpreter if needed. With consent, these will be noted and digitally-recorded.

Data analyses

RCT

Baseline characteristics and important parameters such as follow-up will be summarised and compared between the two study arms using numbers and percentages for categorical data and summary measures of mean or median and spread for continuous data. Being a feasibility RCT, it is not adequately powered to detect a difference in outcomes between the two study arms. However, appropriate regression methods will be used to get initial estimates of effects with confidence intervals to guide the design of the main RCT. All primary analyses will be based on the intention-to-treat principle and will be unadjusted. Subsequently, the adjustment will be done for the baseline data and site. No interim analysis is planned.

Qualitative evaluation

For peer review only - http://bmjopen.bmj.com/site/about/guidelines.xhtml

All the semi-structured interviews will be transcribed (verbatim), translated (if necessary), anonymised and checked for accuracy. An interpretive analysis will be conducted using thematic analysis, using NVivo software. Transcripts will be read and re-read by two qualitative researchers. These researchers will develop the initial codes and will apply initially to a small number of transcripts, enabling further iteration of the thematic index. We will use illustrative nonattributable quotations [27].

Patient and public involvement

The research topic was identified and discussed with a Public Engagement Coordinator and among a patient and public involvement group. They acknowledged the importance of this research topic and the issues identified during these discussions were taken into consideration while designing the study. They are involved in the discussions and are giving feedback on different aspects of the study.

Ethics and dissemination

Ethics and other related issues

Ethics approval has been obtained from the following Research Ethics Committees: Faculty of Medicine and Health Sciences, University of Nottingham, UK (14-1805); CCDC, India (CCDC_IEC_09_2018); BNCHY, India (BNCHY/IEC/2/2019) and S-VYASA, India (RES/IEC-SVYASA/138/2018). Written informed consent is obtained from all the participants. We have also received approval from the Health Ministry's Screening Committee (HMSC, India). An independent Trial Steering Committee (TSC) is monitoring and providing overall supervision for the study.

Serious adverse events

Like other physical activities, Yoga is known to be safe [10]. Information will be collected on serious adverse events (including death) occurring in participants that may be attributed to the

For peer review only - http://bmjopen.bmj.com/site/about/guidelines.xhtml

interventions. Based on medical and scientific judgement, an independent clinician will determine the relationship of any event to the interventions.

Participant withdrawal

Participants will be withdrawn from the study either at their request or at the discretion of the site investigator e.g., if diagnosed with diabetes (will receive the standard treatment) or if no longer safe to participate in physical activities (determined by PAR-Q/clinician) [29]. They will be made aware that this will not affect their future care. Also, they will be made aware (via the participant information sheet and consent form) that should they withdraw, the data collected to date will not be erased and may still be used in the final analyses.

Dissemination

The results will be reported according to the relevant extension of the Consolidated Standards of Reporting Trials (CONSORT) statement i.e., for randomised pilot and feasibility trials [30]. The results will be widely disseminated among key stakeholders through various avenues, such as through dissemination meetings and informal discussions with them, presentations at national and international conferences, publications in peer-reviewed open-access journals, and press offices and websites of host institutions.

Discussion

We are now conducting a multi-centre feasibility RCT in India to determine the feasibility of undertaking the main RCT. The study started in May 2019, and we are aiming to finish the study by the end of October 2020. If the feasibility is promising (such as recruitment, randomisation, intervention adherence and follow-up), then the parameters estimated will be used to design the main RCT. Decisions over whether to modify the protocol will be informed by the process evaluation, including the qualitative data.

If the intervention is found to be effective in the main RCT, it will be a low-cost, acceptable and local solution to prevent T2DM among high risk people in India and to become healthier

For peer review only - http://bmjopen.bmj.com/site/about/guidelines.xhtml

overall. The future clinical, personal and economic burden of T2DM on patients, their families, the health system and the economy will be prevented. The benefits of preventing T2DM may extend to the prevention of its complications. People will be provided with more evidence-based choices for preventing T2DM. The programme will simultaneously empower them to manage their health. Apart than India and neighbouring South Asian countries, Yoga is popular or becoming popular in many other countries [31,32]. Given that T2DM prevention is a global concern and costs are a concern everywhere, a low-cost Yoga-based T2DM prevention option will be of interest in other countries, particularly in other South Asian countries and in countries with South Asian ethnic minorities.

Declarations

Consent for publication

Not applicable

Availability of data and materials

Not applicable

Competing interests

The authors declare that they have no competing interests.

Funding

The study is funded by the UK's DFID/MRC/NIHR/Wellcome Trust Joint Global Health Trials (MR/R018278/1). The funding agencies have no role in designing the study or in writing the manuscript.

Authors' contributions

KC conceptualised and designed the study with the help of TH, MH, SMG, SAL, NKM, DRH, NT, SK and DP. KC wrote the first draft of the manuscript. PM, KS, TH, MH, SMG, SAL, NKM,

For peer review only - http://bmjopen.bmj.com/site/about/guidelines.xhtml

RN, SM, DRH, NT, SK and DP contributed significantly to the revision of the manuscript. All authors read and approved the final manuscript.

Acknowledgements

The authors would like to extend thanks to all who have participated in this study and the TSC members.

References

1. International Diabetes Federation (IDF). IDF diabetes atlas. 9th Ed. Brussels: IDF. 2019.
2. Anjana RM, Pradeepa R, Deepa M, et al. Prevalence of diabetes and prediabetes in urban and rural India: phase I results of the ICMR-INDIAB study. Diabetologia. 2011;54:3022-7.
3. National Institute for Health and Care Excellence (NICE). Preventing type 2 diabetes: risk identification and interventions for individuals at high risk. Manchester: NICE. 2012.
4. Gong QH, Kang JF, Ying YY, et al. Lifestyle interventions for adults with impaired glucose tolerance: a systematic review and meta-analysis of the effects on glycemic control. Intern Med. 2015;54:303-10.
5. World Health Organization (WHO). Global strategy on diet, physical activity and health. Geneva: WHO. 2015.
6. Anjana RM, Pradeepa R, Das AK, et al. Physical activity and inactivity patterns in India: results from the ICMR-INDIAB study (Phase-1) [ICMR-INDIAB-5]. Int J Behav Nutr Phys Act. 2014;11:26.
7. Gulati S, Misra A, Sharma M. Dietary fats and oils in India. Curr Diabetes Rev. 2017;13(5):438-43.
8. Minocha S, Thomas T, Kurpad AV. Are 'fruits and vegetables' intake really what they seem in India? Eur J Clin Nutr. 2018;72:603-8.
9. Feuerstein G. The deeper dimensions of Yoga: theory and practice. Boston: Shambhala Publications. 2003.

For peer review only

10. National Health Service (NHS). A guide to Yoga: exercise. UK: NHS. 2018. https://www.nhs.uk/live-well/exercise/guide-to-yoga/ accessed on 2 Sep 2019.

11. Anderson JG, Taylor AG. The metabolic syndrome and mind-body therapies: a systematic review. J Nutr Metab. 2011;2011:276419.

12. Anjana RM, Ranjani H, Unnikrishnan R, et al. Exercise patterns and behaviour in Asian Indians: data from the baseline survey of the Diabetes Community Lifestyle Improvement Program. Diabetes Res Clin Pract. 2015;107:77-84.

13. Centers for Disease Control and Prevention (CDC) and American College of Sports Medicine (ACSM). General physical activities defined by level of intensity. Atlanta: CDC. 2015.

14. Sinha B, Ray US, Pathak A, et al. Energy cost and cardiorespiratory changes during the practice of Surya Namaskar. Indian J Physiol Pharmacol. 2004;48:184-90.

15. Mody BS. Acute effects of Surya Namaskar on the cardiovascular and metabolic system. J Bodyw Mov Ther. 2011;15:343-7.

16. Cramer H, Innes KE, Michalsen A, et al. Yoga for adults with type 2 diabetes mellitus (protocol). Cochrane Database Syst Rev. 2015;4:CD011658.

17. Cramer H, Lauche R, Haller H, et al. Effects of Yoga on cardiovascular disease risk factors: a systematic review and meta-analysis. Int J Cardiol. 2014;173:170-83.

18. Cramer H, Langhorst J, Dobos G, et al. Yoga for metabolic syndrome: a systematic review and meta-analysis. Eur J Prev Cardiol. 2016;23:1982-93.

19. Kumar V, Jagannathan A, Philip M, et al. Role of Yoga for patients with type II diabetes mellitus: a systematic review and meta-analysis. Complement Ther Med. 2016;25:104-12.

20. Cui J, Yan JH, Yan LM, et al. Effects of Yoga in adults with type 2 diabetes mellitus: a meta-analysis. J Diabetes Investig. 2017;8:201-9.

21. Medical Research Council (MRC). Developing and evaluating complex interventions. London: MRC. 2008.

22. Samal J. Situational analysis and future directions of AYUSH: an assessment through 5-year plans of India. J Intercult Ethnopharmacol. 2015;4:348-54.

For peer review only

For peer review only - http://bmjopen.bmj.com/site/about/guidelines.xhtml

23. National Institute for Health Research (NIHR). Feasibility and pilot studies. UK: NIHR. 2014.

24. Ranjani H, Weber MB, Anjana RM, et al. Recruitment challenges in a diabetes prevention trial in a low- and middle-income setting. Diabetes Res Clin Pract. 2015;110:51-9.

25. Hooper R. Justifying sample size for a feasibility study. UK: NIHR. 2014.

26. Dettori JR. Loss to follow-up. Evid Based Spine Care J. 2011;2:7-10.

27. Green J, Thorogood N. Qualitative methods for health research. 3rd Ed. London: SAGE. 2014.

28. Buysschaert M, Medina JL, Buysschaert B, et al. Definitions (and current controversies) of diabetes and prediabetes. Curr Diabetes Rev. 2016;12:8-13.

29. Adams R. Revised physical activity readiness questionnaire. Can Fam Physician. 1999;45:992, 995, 1004-5.

30. Eldridge SM, Chan CL, Campbell MJ, et al. CONSORT 2010 statement: extension to randomised pilot and feasibility trials. BMJ. 2016;355:i5239.

31. Birdee GS, Legedza AT, Saper RB, et al. Characteristics of Yoga users: results of a national survey. J Gen Intern Med. 2008;23:1653-8.

32. Ding D, Stamatakis E. Yoga practice in England 1997-2008: prevalence, temporal trends, and correlates of participation. BMC Res Notes. 2014;7:172.

33. Craig CL, Marshall AL, Sjöström M, et al. International physical activity questionnaire: 12-country reliability and validity. Med Sci Sports Exerc. 2003;35:1381-95.

34. Herdman M, Gudex C, Lloyd A, et al. Development and preliminary testing of the new five-level version of EQ-5D (EQ-5D-5L). Qual Life Res. 2011;20:1727-36.

35. Henry JD, Crawford JR. The short-form version of the depression anxiety stress scales (DASS-21): construct validity and normative data in a large non-clinical sample. Br J Clin Psychol. 2005;44:227-39.

36. Bandura A. Guide for constructing self-efficacy scales. In: Pajares F, Urdan T (Editors). Self-efficacy beliefs of adolescents. Greenwich: Information Age Publishing. 2006;307-37.

For peer review only

For peer review only - http://bmjopen.bmj.com/site/about/guidelines.xhtml

Figure 1: RCT design

[Insert]

For peer review only

For peer review only - http://bmjopen.bmj.com/site/about/guidelines.xhtml

Table 1: Data collection

	Face-to-face assessments*			
	Assessment details	Screening and recruitment	Baseline	Final at 6-month
**Eligibility assessment**		√		
**Socio-demographics**			√	
**Medical and surgical history**			√	
**Family history of diabetes**			√	
**Current medications**			√	√
**Biochemical parameters^**				
Blood glucose				
Fasting plasma glucose	Glucose oxidase-peroxidase (GOD-POD) method		√	√
Glycated haemoglobin (HbA1c)	High-performance liquid chromatography (HPLC) method		√	√
Lipid profile				
Total cholesterol	Cholesterol oxidase method		√	√
High-density lipoprotein (HDL)	Direct clearance method		√	√
Low-density lipoprotein (LDL)	Direct clearance method		√	√
Very low-density lipoprotein (VLDL)	Calculated value		√	√
Triglyceride	Lipase/Glycerol-3-phosphate oxidase-phenol+aminophenazone (GPO-PAP) no correction method		√	√
**Physiological parameters**				
Blood pressure	Omron HEM-7201		√	√
Heart rate	Omron HEM-7201		√	√
**Anthropometric parameters**				
Waist circumference	Seca 201 (measuring tape)		√	√
Weight	Omron HN-286 (weighing scale)		√	√
Height	Seca 206 (stadiometer)		√	√
Body mass index (BMI)	Calculated value		√	√
**Diet**	Time-recall: past 1-week		√	√

For peer review only

For peer review only - http://bmjopen.bmj.com/site/about/guidelines.xhtml

**Physical activity**	International physical activity questionnaire (IPAQ) – short; time-recall: past 1-week[33]		√	√
**Tobacco usage**			√	√
**Alcohol consumption**			√	√
**Health-related quality-of-life**	EuroQol-5D-5L (EQ-5D-5L); time-recall: at the time of questionnaire completion[34]		√	√
**Depression, anxiety and stress**	Depression, anxiety and stress scale (DASS-21); time recall: past 1-week[35]		√	√
**Yoga practice**	Time-recall: past 1-week		√	√
**Self-efficacy** (to assess confidence in participant's ability to practise Yoga)	0-100 rating scale; time-recall: at the time of questionnaire completion[36]		√	√

*A standard operating procedure has been developed for this purpose.
^Blood samples are analysed at the International Organization for Standardization (ISO) or Christian Medical College External Quality Assurance Scheme (Vellore, India) accredited laboratories.

For peer review only

Table 2: Structure of YOGA-DP

Week	Group Yoga sessions delivered by YOGA-DP instructors	Self-practice of Yoga at home using YOGA-DP booklet and a video	Extra features
1-4 (month 1)	At least two sessions of 45 minutes per week. An attendance register is kept.	--	At the first session, the instructor is giving participants part one of our programme booklet. This gives them information about being at high risk of T2DM and how to prevent T2DM (i.e., by being more physically active, keeping a healthy weight, eating less fat (especially saturated fat) and eating more fibre).
5-12 (month 2-3)	At least two sessions of 75 minutes per week. An attendance register is kept.	--	At the last session, the instructor is giving participants part two of our programme booklet and a video. These give them information on Yoga practice to prevent T2DM. Also, a Yoga diary and non-slippery Yoga mat are provided for self-practice of Yoga at home.
13-24 (month 4-6)	At least one session of 75 minutes every four weeks. An attendance register is kept.	At least two sessions of 75 minutes per week. Participants are given the Yoga diary to record their Yoga practice (types and minutes).	The instructor is phoning participants every week to offer support and help and to troubleshoot any problems.
25+ (month 7+)	--	At least two sessions of 75 minutes per week. Participants are given the Yoga diary to record their Yoga practice (types and minutes).	--

For peer review only - http://bmjopen.bmj.com/site/about/guidelines.xhtml

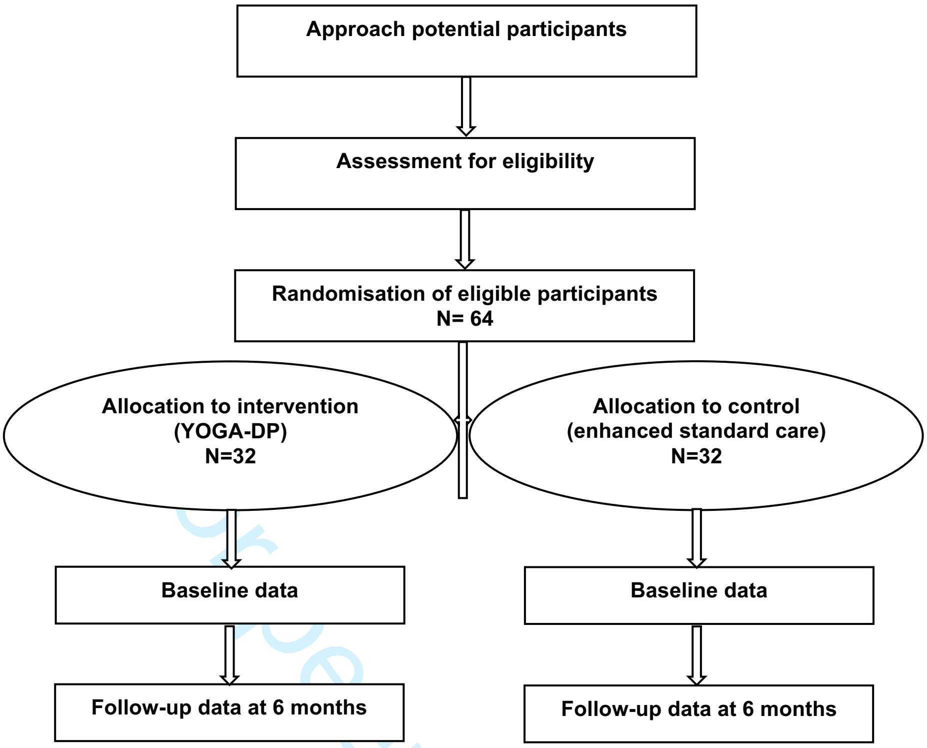
Approach potential participants
Assessment for eligibility
Randomisation of eligible participants
N= 64
Allocation to intervention
(YOGA-DP)
N=32
Allocation to control
(enhanced standard care)
N=32
Baseline data
Baseline data
Follow-up data at 6 months
Follow-up data at 6 months

Table S1: Structure and content of the Yoga sessions

Yogic practices	Week 1-4<br><br>Each session will last for 45 minutes with the time split as follows:	Week 5+<br><br>Each session should last for 75 minutes with the time split as follows:	Details
Shithilikarana Vyayama	Around 5 minutes	Around 5 minutes	(1) Neck rotation *30 seconds*<br>(2) Shoulder rotation *30 seconds*<br>(3) Elbow flexion and extension *30 seconds*<br>(4) Wrist rotation *30 seconds*<br>(5) Finger movement *30 seconds*<br>(6) Waist rotation *30 seconds*<br>(7) Knee flexion and extension *1 minute*<br>(8) Ankle rotation *1 minute*<br>(9) Toe movement *30 seconds*
Surya Namaskar	--	Around 15 minutes	The below mentioned 12 steps constitute one set of Surya Namaskar. To complete one round of Surya Namaskar, participants need to repeat these 12 steps on the other side of their body (i.e., by extending their left leg behind in step number 4 and bringing their left leg forward in step number 9). Initially, they should practise Surya Namaskar at a slower pace. Only with practice over some time, they may try to do 12 rounds of it at a faster pace for around 15 minutes (i.e., a couple of seconds per step).<br>(1) Pranamasana (prayer pose)<br>(2) Hastauttanasana (raised arms pose)<br>(3) Padahastasana (hands to feet pose)<br>(4) Ashwa Sanchalanasana (equestrian pose)<br>(5) Dandasana (stick pose)<br>(6) Ashtanga Namaskara Asana (salute with eight parts)<br>(7) Bhujangasana (cobra pose)<br>(8) Parvatasana (mountain pose)<br>(9) Ashwa Sanchalanasana (equestrian pose)<br>(10) Padahastasana (hands to feet pose)<br>(11) Hastauttanasana (raised arms pose)<br>(12) Pranamasana (prayer pose)
Asana	Around 15 minutes	Around 30 minutes	Two-sided poses (right and left) are to be practised for about 3 minutes (1.5 minutes on each side) and central-positioned poses are to be practised for about 1.5 minutes. In each session, the Yogic poses are selected from the list below to prevent boredom from the similarity of routine. Advanced Yogic poses are introduced from week 5 onwards, for example, Konasana (angle pose), Trikonasana (triangle pose), Paravakonasana (lateral angle pose), Ardhaustrasana (half camel pose), Ustrasana (camel pose), Dhanurasana (bow pose) and Naukasana (boat pose).<br>(A) Standing poses

For peer review only

			(1) Tadasana (palm tree pose) *1.5 minutes*<br>(2) Ardhachakrasana (half wheel pose) *1.5 minutes*<br>(3) Katichakrasana (waist wheel pose) *3 minutes*<br>(4) Konasana (angle pose) or Trikonasana (triangle pose) or Paravakonasana (lateral angle pose): alternatively *3 minutes*<br>(B) Sitting poses<br>(1) Vajrasana (adamant pose) *1.5 minutes*<br>(2) Mandukasana (frog pose) *1.5 minutes*<br>(3) Ardhaustrasana (half camel pose) or Ustrasana (camel pose): alternatively *1.5 minutes*<br>(4) Vakrasana (twisted pose) or Ardhamatsyendrasana (half spinal twist pose): alternatively *3 minutes*<br>(5) Paschimottanasana (seated forward bend pose) or Janusirsasana (head to knee pose): alternatively *1.5 minutes or 3 minutes, respectively*<br>(C) Lying poses- front/prone<br>(1) Ardhashalabhasana (half locust pose) or Poornashalabhasana (full locust pose): alternatively *3 minutes or 1.5 minutes, respectively*<br>(2) Dhanurasana (bow pose) *1.5 minutes*<br>(3) Makarasana (crocodile pose) *1.5 minutes*<br>(D) Lying poses- back/supine<br>(1) Uttanapadasana (raised legs pose) or Ardhahalasana (half plough pose): alternatively *1.5 minutes*<br>(2) Pavanamuktasana (wind relieving pose) *1.5 minutes*<br>(3) Naukasana (boat pose) *1.5 minutes*<br>(4) Saralmatsyasana (easy fish pose) *1.5 minutes*
Pranayama	Around 13 minutes	Around 13 minutes	(1) Vibhagiya Pranayama (sectional breathing) *4 minutes*<br>(2) Nadishodhana Pranayama (alternate nostril breathing) *3 minutes*<br>(3) Kapalbhati Pranayama (skull shining breathing) or Bhastrika Pranayama (bellow breathing): alternately *3 minutes*<br>(4) Bhramari Pranayama (bee breathing) *3 minutes*
Dhyana and relaxation practices	Around 12 minutes	Around 12 minutes	In each session, the following Dhyana and relaxation practices are to be done in a darkened room.<br>(1) A Kara chanting, U Kara chanting and M Kara chanting *3 minutes*<br>(2) Yoga Nidra (Yogic sleep) *9 minutes*

For peer review only

STANDARD PROTOCOL ITEMS: RECOMMENDATIONS FOR INTERVENTIONAL TRIALS

SPIRIT 2013 Checklist: Recommended items to address in a clinical trial protocol and related documents*

Section/item	Item No	Description	Addressed on page number
**Administrative information**			
Title	1	Descriptive title identifying the study design, population, interventions, and, if applicable, trial acronym	1
Trial registration	2a	Trial identifier and registry name. If not yet registered, name of intended registry	3
	2b	All items from the World Health Organization Trial Registration Data Set	3
Protocol version	3	Date and version identifier	Not available in web format, please use the contact details to request a copy
Funding	4	Sources and types of financial, material, and other support	15
Roles and responsibilities	5a	Names, affiliations, and roles of protocol contributors	1
	5b	Name and contact information for the trial sponsor	15
	5c	Role of study sponsor and funders, if any, in study design; collection, management, analysis, and interpretation of data; writing of the report; and the decision to submit the report for publication, including whether they will have ultimate authority over any of these activities	15

For peer review only

	5d	Composition, roles, and responsibilities of the coordinating centre, steering committee, endpoint adjudication committee, data management team, and other individuals or groups overseeing the trial, if applicable (see Item 21a for data monitoring committee)	Not available in web format, please use the contact details to request a copy
**Introduction**			
Background and rationale	6a	Description of research question and justification for undertaking the trial, including summary of relevant studies (published and unpublished) examining benefits and harms for each intervention	4-6
	6b	Explanation for choice of comparators	9
Objectives	7	Specific objectives or hypotheses	4-6
Trial design	8	Description of trial design including type of trial (eg, parallel group, crossover, factorial, single group), allocation ratio, and framework (eg, superiority, equivalence, noninferiority, exploratory)	6
**Methods: Participants, interventions, and outcomes**			
Study setting	9	Description of study settings (eg, community clinic, academic hospital) and list of countries where data will be collected. Reference to where list of study sites can be obtained	6
Eligibility criteria	10	Inclusion and exclusion criteria for participants. If applicable, eligibility criteria for study centres and individuals who will perform the interventions (eg, surgeons, psychotherapists)	7-9
Interventions	11a	Interventions for each group with sufficient detail to allow replication, including how and when they will be administered	8-9
	11b	Criteria for discontinuing or modifying allocated interventions for a given trial participant (eg, drug dose change in response to harms, participant request, or improving/worsening disease)	12
	11c	Strategies to improve adherence to intervention protocols, and any procedures for monitoring adherence (eg, drug tablet return, laboratory tests)	8-9

For peer review only - http://bmjopen.bmj.com/site/about/guidelines.xhtml

	11d	Relevant concomitant care and interventions that are permitted or prohibited during the trial	7______________
Outcomes	12	Primary, secondary, and other outcomes, including the specific measurement variable (eg, systolic blood pressure), analysis metric (eg, change from baseline, final value, time to event), method of aggregation (eg, median, proportion), and time point for each outcome. Explanation of the clinical relevance of chosen efficacy and harm outcomes is strongly recommended	9-10,19___________
Participant timeline	13	Time schedule of enrolment, interventions (including any run-ins and washouts), assessments, and visits for participants. A schematic diagram is highly recommended (see Figure)	19-20______________
Sample size	14	Estimated number of participants needed to achieve study objectives and how it was determined, including clinical and statistical assumptions supporting any sample size calculations	10-11______________
Recruitment	15	Strategies for achieving adequate participant enrolment to reach target sample size	6______________
**Methods: Assignment of interventions (for controlled trials)**			
Allocation:			
Sequence generation	16a	Method of generating the allocation sequence (eg, computer-generated random numbers), and list of any factors for stratification. To reduce predictability of a random sequence, details of any planned restriction (eg, blocking) should be provided in a separate document that is unavailable to those who enrol participants or assign interventions	7-8______________
Allocation concealment mechanism	16b	Mechanism of implementing the allocation sequence (eg, central telephone; sequentially numbered, opaque, sealed envelopes), describing any steps to conceal the sequence until interventions are assigned	7-8______________
Implementation	16c	Who will generate the allocation sequence, who will enrol participants, and who will assign participants to interventions	7-8______________
Blinding (masking)	17a	Who will be blinded after assignment to interventions (eg, trial participants, care providers, outcome assessors, data analysts), and how	7-8______________
	17b	If blinded, circumstances under which unblinding is permissible, and procedure for revealing a participant's allocated intervention during the trial	N/A_____________

Methods: Data collection, management, and analysis

Data collection methods	18a	Plans for assessment and collection of outcome, baseline, and other trial data, including any related processes to promote data quality (eg, duplicate measurements, training of assessors) and a description of study instruments (eg, questionnaires, laboratory tests) along with their reliability and validity, if known. Reference to where data collection forms can be found, if not in the protocol	19-20
	18b	Plans to promote participant retention and complete follow-up, including list of any outcome data to be collected for participants who discontinue or deviate from intervention protocols	12
Data management	19	Plans for data entry, coding, security, and storage, including any related processes to promote data quality (eg, double data entry; range checks for data values). Reference to where details of data management procedures can be found, if not in the protocol	Not available in web format, please use the contact details to request a copy
Statistical methods	20a	Statistical methods for analysing primary and secondary outcomes. Reference to where other details of the statistical analysis plan can be found, if not in the protocol	11
	20b	Methods for any additional analyses (eg, subgroup and adjusted analyses)	11
	20c	Definition of analysis population relating to protocol non-adherence (eg, as randomised analysis), and any statistical methods to handle missing data (eg, multiple imputation)	11

Methods: Monitoring

Data monitoring	21a	Composition of data monitoring committee (DMC); summary of its role and reporting structure; statement of whether it is independent from the sponsor and competing interests; and reference to where further details about its charter can be found, if not in the protocol. Alternatively, an explanation of why a DMC is not needed	Not available in web format, please use the contact details to request a copy
	21b	Description of any interim analyses and stopping guidelines, including who will have access to these interim results and make the final decision to terminate the trial	N/A
Harms	22	Plans for collecting, assessing, reporting, and managing solicited and spontaneously reported adverse events and other unintended effects of trial interventions or trial conduct	12

Auditing	23	Frequency and procedures for auditing trial conduct, if any, and whether the process will be independent from investigators and the sponsor	Not available in web format, please use the contact details to request a copy
**Ethics and dissemination**			
Research ethics approval	24	Plans for seeking research ethics committee/institutional review board (REC/IRB) approval	11-12
Protocol amendments	25	Plans for communicating important protocol modifications (eg, changes to eligibility criteria, outcomes, analyses) to relevant parties (eg, investigators, REC/IRBs, trial participants, trial registries, journals, regulators)	Not available in web format, please use the contact details to request a copy
Consent or assent	26a	Who will obtain informed consent or assent from potential trial participants or authorised surrogates, and how (see Item 32)	6-7
	26b	Additional consent provisions for collection and use of participant data and biological specimens in ancillary studies, if applicable	Not available in web format, please use the contact details to request a copy
Confidentiality	27	How personal information about potential and enrolled participants will be collected, shared, and maintained in order to protect confidentiality before, during, and after the trial	Not available in web format, please use the contact details to request a copy
Declaration of interests	28	Financial and other competing interests for principal investigators for the overall trial and each study site	14
Access to data	29	Statement of who will have access to the final trial dataset, and disclosure of contractual agreements that limit such access for investigators	Not available in web format, please use the contact details to request a copy

For peer review only

Ancillary and post-trial care	30	Provisions, if any, for ancillary and post-trial care, and for compensation to those who suffer harm from trial participation	Not available in web format, please use the contact details to request a copy
Dissemination policy	31a	Plans for investigators and sponsor to communicate trial results to participants, healthcare professionals, the public, and other relevant groups (eg, via publication, reporting in results databases, or other data sharing arrangements), including any publication restrictions	14
	31b	Authorship eligibility guidelines and any intended use of professional writers	N/A
	31c	Plans, if any, for granting public access to the full protocol, participant-level dataset, and statistical code	Not available in web format, please use the contact details to request a copy
**Appendices**			
Informed consent materials	32	Model consent form and other related documentation given to participants and authorised surrogates	Attached as supplementary files
Biological specimens	33	Plans for collection, laboratory evaluation, and storage of biological specimens for genetic or molecular analysis in the current trial and for future use in ancillary studies, if applicable	Not available in web format, please use the contact details to request a copy

*It is strongly recommended that this checklist be read in conjunction with the SPIRIT 2013 Explanation & Elaboration for important clarification on the items. Amendments to the protocol should be tracked and dated. The SPIRIT checklist is copyrighted by the SPIRIT Group under the Creative Commons "Attribution-NonCommercial-NoDerivs 3.0 Unported" license.

For peer review only
